# Supplementary material for: Pharmacoproteomic characterisation of human colon and rectal cancer
Source: Mol Syst Biol. 2017 Nov 3;13(11):951. doi: 10.15252/msb.20177701 (PMC5731344; doi:10.15252/msb.20177701)
Supplement: Supplementary file 1 — Appendix [file MSB-13-951-s001.pdf]

Pharmacoproteomic characterisation of human colon and rectal cancer

Martin Frejno[1,2], Riccardo Zenezini Chiozzi[2,3], Mathias Wilhelm[2], Heiner Koch[2,4,5], Runsheng Zheng[2], Susan Klaeger[2,4,5], Benjamin Ruprecht[2,6], Chen Meng[2], Karl Kramer[2], Anna Jarzab[2], Stephanie Heinzlmeir[2,4,5], Elaine Johnstone[1], Enric Domingo[1,7], David Kerr[8], Moritz Jesinghaus[9], Julia Slotta-Huspenina[9], Wilko Weichert[9], Stefan Knapp[10], Stephan M Feller[11,12,*], Bernhard Kuster[2,4,6,13,*]

[1]Department of Oncology, University of Oxford, Oxford OX3 7DQ, United Kingdom
[2]Chair of Proteomics and Bioanalytics, Technical University of Munich, Freising 85354, Germany
[3]Chemistry Department, University of Rome, La Sapienza, Rome 00185, Italy
[4]German Cancer Consortium (DKTK), Munich 85354, Germany
[5]German Cancer Research Center (DKFZ), Heidelberg 69120, Germany
[6]Center for Integrated Protein Science (CIPSM), Munich 81377, Germany
[7]Wellcome Trust Centre for Human Genetics (WTCHG), University of Oxford, Oxford OX3 7BN, United Kingdom
[8]Nuffield Division of Clinical Laboratory Sciences (NDCLS), University of Oxford, Oxford OX3 9DU, United Kingdom
[9]Institute of Pathology, Technical University of Munich, Munich 81675, Germany
[10]Institute of Pharmaceutical Chemistry, Goethe University, Frankfurt am Main 60439, Germany
[11]Weatherall Institute of Molecular Medicine, University of Oxford, Oxford OX3 9DS, United Kingdom
[12]Institute of Molecular Medicine, Martin-Luther-University, Halle 06120, Germany
[13]Bavarian Biomolecular Mass Spectrometry Center (BayBioMS), Freising 85354, Germany
[*]Corresponding author

Correspondence

kuster@tum.de

stephan.feller@uk-halle.de

Final character count including spaces: 76,048

Appendix Supplementary Methods Error! Bookmark not defined.
Quantification of LC-MS/MS raw data 3
giBAQ quantification 3
LFQ quantification 4
Post-processing of quantitative LC-MS/MS data 5
Full Proteomes 5
Kinobeads 5
Reanalysis of public mRNA datasets 6
Pre-processing 6
Combination of transcriptomics datasets 10
Multi-omics data integration strategy 12
Computation of protein/mRNA fold-changes 12
Integration of transcriptomics and proteomics 12
mRNA-guided missing value imputation 13
Minimum-guided missing value imputation 14
Combination of the CRC65 and CPTAC datasets 16
Identification & characterisation of subtypes 16
Identification of subtypes 16
Differential expression analysis 17
Functional annotation enrichment & clustering 18
Reanalysis of public dose-response datasets 19
Pre-processing 19
Dose-response models & parameter extraction 20
Modelling of Drug Sensitivity 21
Cox proportional hazards regression 24
Annotation information 25

Appendix Supplementary References **26**

Appendix Supplementary Methods

Quantification of LC-MS/MS raw data

giBAQ quantification

For full proteomes, we used unique and razor peptides for quantification. MaxQuant was set to calculate iBAQ intensities (Schwanhausser et al, 2011), however we instead used a modified version of the classical iBAQ approach termed giBAQ to estimate absolute quantities of gene groups as opposed to protein groups, excluding reverse and contaminant hits from the proteinGroups.txt output of MaxQuant. Unless otherwise stated, the CPTAC and CRC65 datasets were processed separately from one another. In order to ensure comparability with transcriptomics data, we assigned every protein group to a single gene group using the following heuristic with four mapping groups (MGs): First, we retrieved a mapping of Uniprot identifiers in the "Majority protein IDs" column to HGNC symbols from Ensembl using biomaRt v2.25.2, collapsing isoforms to their parent Uniprot identifier. This resulted in two mapping groups, one for the Swiss-Prot entries (MG1) and one for the TrEMBL entries (MG2) contained in Uniprot, which were subsequently filtered to remove Swiss-Prot and TrEMBL identifiers mapping to more than one HGNC symbol. For each protein group, we then selected the first Swiss-Prot entry in the "Majority protein IDs" column to represent this protein group and subsequently assigned it the corresponding HGNC symbol from MG1. Afterwards, protein groups left without an HGNC symbol were represented by the first TrEMBL entry in the "Majority protein IDs" column and subsequently assigned the corresponding HGNC symbol from MG2. For protein groups still left without an HGNC symbol, we then used the R package Uniprot.ws v2.10.2 to retrieve a mapping of Uniprot identifiers in the "Majority protein IDs" column to HGNC symbols from Uniprot (MG3), collapsing isoforms to their parent Uniprot identifier. Protein groups left without an HGNC symbol were then represented by the first Uniprot entry in the "Majority protein IDs" column and subsequently assigned the first corresponding HGNC symbol from MG3. Afterwards, all protein groups still left without an HGNC symbol were assigned the first HGNC symbol from the "Gene names" column of the proteinGroups.txt output file (MG4). This heuristic guaranteed that the majority of HGNC-symbol-assignments were done using biomaRt (99% for both the CPTAC and CRC65 datasets), a system which–as opposed to Uniprot.ws or MaxQuant–could also be used to map HGNC symbols to the various identifiers encountered in the transcriptomics datasets described below, ensuring maximum comparability of transcriptomics and proteomics data. We favoured MG1 over MG2 during assignment of HGNC symbols, since it was based on Swiss-Prot entries, which are manually curated. Selecting the first Swiss-Prot (MG1), TrEMBL

(MG2) or Uniprot (MG3) entry in the "Majority protein IDs" column to represent a given protein group during HGNC-symbol-assignment in their respective mapping group further made sure that these assignments were always based on the identifier corresponding to the protein with the highest number peptides in the respective protein group. It is worth noting that 96% and 97% of our heuristic HGNC-symbol assignments for the CPTAC patient and CRC65 cell line datasets were identical to the corresponding values in the "Gene names" column of the proteinGroups.txt output file, even though it only served as the source for 1% and 0.1% of the HGNC-symbols, respectively.

Next, we extracted the number of theoretical peptides per protein group used by MaxQuant for the calculation of iBAQ intensities as the ratio of raw intensities to iBAQ intensities, rounded to the nearest integer. As expected, these values were constant for each protein group across all samples in the two datasets and were equal to the values determined by *in silico* digestion of the fasta file of UniprotKB used in the database search by MaxQuant with the Protein Digestion Simulator v2.2.5679. Protein groups with only missing intensity values across one of the two datasets or no assigned HGNC-symbol were excluded from the respective dataset. For each gene group as defined by a common HGNC-symbol, we then summed up the raw intensities of protein groups in this gene group in a sample-wise manner and divided them by the sum of the number of theoretical peptides for these proteins, which satisfied the standard iBAQ criteria adapted to our MaxQuant search parameters. These were fully tryptic peptides between 7 and 30 amino acids without missed cleavages, since the minimum peptide length in MaxQuant was set to 7 instead of 6. For a gene group only containing proteins which do not share any theoretical peptides, this quantity termed giBAQ value will be equal to the iBAQ value obtained for a hypothetical protein constructed from them, thereby normalising to the length of the portion of the gene accessible by mass spectrometry, rather than the individual protein lengths.

LFQ quantification

For Kinobeads experiments, we used unique and razor peptides for quantification. MaxQuant was set to calculate LFQ intensities with an LFQ minimum ratio count of two. Fast LFQ was used to determine normalisation factors, with the minimum number of neighbours set to three, the average number of neighbours set to six and the "Stabilize large LFQ ratios" option enabled. We did not require MS2 spectra for pair-wise peptide intensity comparisons during the calculation of LFQ intensities. Gene names associated with protein groups were remapped as described above for full proteome data. For each gene group and sample, we then

summed up the LFQ intensities of protein groups in this gene group to generate a gene-level quantification, which was used in all subsequent analyses.

Post-processing of quantitative LC-MS/MS data

Full Proteomes

The CRC65 and CPTAC datasets were processed separately from one another. For the CPTAC dataset, we restricted our analysis to CPTAC sample IDs not removed in the original publication (Zhang et al, 2014) due to duplication. Following common practice, giBAQ values were log2-transformed and median-centred. Since the CRC65 cell line panel contained two pairs of concordant cell lines (HDC-54/HDC-57 and LS 180/LS 174T; Bracht et al, 2010; Klijn et al, 2015; Medico et al, 2015), we replaced missing values in HDC-54 and LS 180 with measured values from HDC-57 and LS 174T, respectively. HDC-57 and LS 174T were subsequently excluded from the analysis. It is worth noting that we did not just calculate the arithmetic mean of these cell line pairs, since this would prevent minimum-guided missing value imputation on the peptide level with raw-file-specific backgrounds, as described in its respective section.

Kinobeads

Since we observed batch effects between the three different biological replicates, we decided to log2-transform and median-centre them separately from one another, followed by batch-effect removal using ComBat (Johnson et al, 2007). Briefly, we first restricted our dataset to gene groups consistently measured between the different replicates and across the dataset, selecting gene groups with at least two out of three reported LFQ values per cell line in at least 23 cell lines. Next, we removed all gene groups with one or fewer reported LFQ values per replicate in order to eliminate noise and enable subsequent parametric batch adjustment using ComBat, implemented in the sva R package v3.18.0, with MSI status as a covariate as described by Guinney and colleagues (Guinney et al, 2015). We used the median across all three replicates to summarise the LFQ values for each cell line, taking the median across all six replicates for the two pairs of concordant cell lines described above; HDC-54 was selected to represent HDC-54/HDC-57 and LS 180 was selected to represent LS 180/LS 174T.

Reanalysis of public mRNA datasets

Pre-processing

We downloaded 10 mRNA datasets (GSE36133 - Barretina et al, 2012; E-MTAB-783 - Garnett et al, 2012; E-MTAB-2706 - Klijn et al, 2015; GSE28567 - Loboda et al, 2011; GSE59857 - Medico et al, 2015; Supplementary Table S6 - Mouradov et al, 2014; platform codes "IlluminaGA_RNASeq" + "AgilentG4502A_07_3" - TCGA Network, 2012; GSE8332 - Wagner et al, 2007; GSE24795 - Wilding et al, 2010, respectively) and processed them as follows, automatically obtaining the required chip definition files (.cdf) from Bioconductor for microarray datasets, if not stated otherwise. The same cell lines have inconsistent names in the different transcriptomics datasets, which is why the first pre-processing step always involved the mapping of all cell line names to a set of consistent sample names (see also Table EV6A).

GSE36133

We used GEOquery v2.35.6 to download annotation information associated with GSE36133 and obtained raw .CEL files, as well as the "Expression arrays samples info file" from the CCLE portal of the Broad Institute (http://portals.broadinstitute.org/ccle/data/browseData?conversationPropagation=begin, 31 December 2015). The dataset was restricted to cell lines from the large intestine, as well as C32 and Colo 741, which were derived from skin according to the "Expression arrays samples info file". The preproPara() function from the affyPara package v1.29.0 was then used with background correction through RMA, quantile normalisation and probe summarisation through median polish of perfect match (PM) probes to calculate the classical RMA expression measure. Probes with only missing values across the dataset were excluded. Next, we retrieved a mapping of AffyIDs to HGNC symbols from Ensembl using biomaRt and filtered it to remove AffyIDs, which mapped to more than one HGNC symbol. This mapping was then used to assign each AffyID to its corresponding HGNC symbol. AffyIDs we were not able to map this way were excluded from the dataset. This was one of two datasets for which we were not able to first map primary IDs (in this case AffyIDs) to GeneIDs with the default annotation data downloaded from GEO or related sources. For consistency reasons, GeneIDs served as the basis for HGNC symbol assignment during the analysis of other transcriptomics datasets whenever possible. For GSE36133, this resulted in an expression matrix of 36,877 AffyIDs across 63 cell lines.

E-MTAB-783

We obtained raw .CEL files, as well as the corresponding eSet.r and adf.txt files from ArrayExpress (https://www.ebi.ac.uk/arrayexpress/experiments/E-MTAB-783/files/, 31 December 2015) and downloaded the

"gdsc_manova_input_w5.csv" file from the GDSC portal in order to be able to map cell lines to tissues of origin (ftp://ftp.sanger.ac.uk/pub4/cancerrxgene/releases/release-5.0/, 12 March 2016). The dataset was restricted to cell lines from the large intestine, as well as C32 and SW626, which were derived from skin and ovary, respectively, according to the tissue labels in the "gdsc_manova_input_w5.csv" file. The rest of the pre-processing was similar to GSE36133, with the only difference being that we first mapped AffyIDs to Ensembl GeneIDs using the adf.txt file and afterwards assigned each GeneID to an HGNC symbol using biomaRt as described above. This resulted in an expression matrix of 20,106 AffyIDs across 37 cell lines.

E-MTAB-2706

We obtained Supplementary Table 1 from the original publication by Klijn et al., as well as RPKM data for coding genes from the website accompanying the publication (http://research-pub.gene.com/KlijnEtAl2014/, 06 October 2015). Supplementary Table 1 was used to assign cell lines to their tissues of origin and the dataset was subsequently restricted to colorectal cancer cell lines as well as C32, which was derived from skin. RPKM values equal to zero were treated as missing values and GeneIDs only containing missing values were removed from the dataset. Afterwards, we quantile normalised and log2-transformed the data, followed by assigning each GeneID to an HGNC symbol using biomaRt as described above. This resulted in an expression matrix of 20,965 GeneIDs across 56 cell lines.

GSE28567

We obtained annotation information for GSE28567 through GEOquery and downloaded the appropriate custom chip definition file (.cdf) and the corresponding raw .CEL files from NCBI GEO (Edgar et al, 2002; 06 November 2015). The R package makecdfenv v1.46.0 was used to construct an annotation package from the .cdf file, which was subsequently used to relate probes to locations on the microarray. The rest of the pre-processing was similar to GSE36133, with the only difference being that we first mapped the custom probe IDs to Ensembl GeneIDs using the annotation information from GEO and afterwards assigned each GeneID to an HGNC symbol using biomaRt as described above. This resulted in an expression matrix of 33,749 probe IDs across 67 cell lines.

GSE59857

We used GEOquery to obtain a normalised expression set object and annotation information associated with GSE59857 and downloaded non-normalised expression data from NCBI GEO (04 November 2015), as well as further annotation information for Illumina expression arrays from Bioconductor. The R packages limma

v3.26.2 and beadarray v2.19.1 were used for background correction, normalisation and transformation of raw data. We first generated an ExpressionSetIllumina from the expression set object we downloaded from NCBI GEO and replaced the normalised expression data with the non-normalised data. Subsequently, we performed background correction using the backgroundCorrect function from limma, estimating the parameters of the normal-exponential (normexp) convolution model adapted for microarrays other than Affymetrix arrays by Ritchie et al (2007) using the maximum-likelihood estimator developed by Silver et al (2009) with an offset of 10 in order to ensure that expression values were positive after background correction. Afterwards, the corrected expression data were quantile normalised using the normaliseIllumina function from the beadarray package, followed by log2-transformation of the dataset. This method was used in favour of the neqc method described by Shi and colleagues (Shi et al, 2010) after comparison of the distributions of expression values produced by the two algorithms. We restricted the dataset to probes from coding regions, excluding negative control and bad quality probes, but keeping probes associated with GeneIDs contained in the single-sample predictor for the CMS subtypes published by Guinney et al (2015) regardless of probe quality annotation. Ensembl GeneIDs were mapped to HGNC symbols using biomaRt as described above. This resulted in an expression matrix of 27,293 probe IDs across 155 cell lines.

Supplementary Table S6 from Mouradov et al.

We downloaded (05 August 2014) Supplementary Table S6 from the original publication by Mouradov et al (2014), excluded cell line RW2982 from the dataset as the only cell line not present in the CRC65 panel, treated RPKM values equal to zero as missing values and restricted the dataset to RefSeq IDs containing at least one measured value. Subsequently, RPKM values were quantile normalised and log2-transformed, followed by re-mapping RefSeq IDs to HGNC symbols using biomaRt as described above. This resulted in an expression matrix of 29,973 RefSeq IDs across 12 cell lines.

Platform code "IlluminaGA_RNASeq" from TCGA

We used the TCGAbiolinks R package v1.0.4 to obtain and prepare level 3 data associated with COAD/READ tumours and platform code “IlluminaGA_RNASeq”, for which we also had proteomics data from the CPTAC study on colorectal cancer (Zhang et al, 2014) and which were not excluded from this dataset due to duplication as described above (16 December 2015). We removed duplicate rows from the RPKM expression matrix, remapped the TCGA barcodes to the sample names described in Table EV6A, treated RPKM values equal to zero as missing values and restricted the dataset to GeneIDs containing at least one measured value, as described

above. Afterwards, the dataset was log2-transformed and median-centred, followed by re-mapping of GeneIDs to HGNC symbols using biomaRt as described above. This resulted in an expression matrix of 19,670 GeneIDs across 87 tumours.

Platform code "AgilentG4502A_07_3" from TCGA

We again used the TCGAbiolinks R package v1.0.4 to obtain and prepare level 1 data and MAGE-TAB files associated with COAD/READ tumours and platform code "*AgilentG4502A_07_3*", for which we also had proteomics data from the CPTAC study on colorectal cancer (Zhang et al, 2014; 25 January 2016). The corresponding platform design file (.adf files) was downloaded from the TCGA portal at https://tcga-data.nci.nih.gov/docs/publications/tcga/platformdesign.html (26 January 2016). We used limma for the processing of txt files generated from microarray scans using Agilent's Feature Extraction Software (TCGA Network, 2011). Briefly, an RGList was generated, followed by annotation of the probes using the platform design file in order to perform quality control through annotated MA-plots, boxplots of background intensities, image plots of foreground and background intensities, density plots and the QC metrics calculated by the R package arrayQualityMetrics v3.26.1. Subsequently, we performed background correction using the backgroundCorrect function from limma with the method described by Edwards (2003) and an offset of 50, since it produced the most stable results as judged by inspection of MA-plots following within-array loess normalisation with standard parameters for several background correction methods. Afterwards, between-array normalisation using the "Gquantile" method implemented in the normalizeBetweenArrays function of limma was carried out under the assumption that the signal distributions of the green channels across all arrays should be the same, since the green channel always contained the Universal Human Reference RNA (TCGA Network, 2012). We took the median of replicate probes, filtered out probes without GeneID annotation and finally mapped the GeneIDs to HGNC symbols using biomaRt as described above. This resulted in an expression matrix of 66,728 Agilent IDs across 73 tumours.

GSE8332

We used GEOquery to download annotation information associated with GSE8332 and also obtained raw .CEL files from NCBI GEO (20 November 2015). The dataset was pre-processed similarly to GSE36133 and subsequently restricted to CRC cell lines based on Figure 1 of the original publication (Wagner et al, 2007), as well as C32 and Colo 741, which were derived from skin as described above. It is worth noting that the authors categorised Colo 741 as a CRC cell line, which contradicts the annotations found in the other mRNA datasets

analysed thus far. Using the annotation information downloaded from GEO, AffyIDs were then annotated with GeneIDs, which were subsequently mapped to HGNC symbols using biomaRt as described above. This resulted in an expression matrix of 39,546 AffyIDs across 36 cell lines.

GSE24795

Raw data for GSE24795 were obtained (05 November 2015) and pre-processed as described for GSE8332, with the only difference that no samples needed to be omitted, since all cell lines included in the study were CRC cell lines. This resulted in an expression matrix of 39,546 AffyIDs across 30 cell lines.

Combination of transcriptomics datasets

We aggregated the different transcriptomics datasets described above into one expression matrix using a scheme similar to the one published by Guinney and colleagues (Guinney et al, 2015). As mentioned in the original publication, we expected strong batch effects between the different transcriptomics datasets and also needed to select a single probe or primary ID to represent the expression of a given gene in each dataset in order to avoid inconsistent mRNA quantification. But since we intended to compare the abundance of as many mRNA species to as many protein species across as many samples as possible, respectively, we refrained from removing outlier samples from each dataset separately and also did not restrict the list of quantifiable mRNA species to the ones with the largest median absolute deviation (MAD). We also did not select a reference dataset *a priori*, but rather computed similar consistency measures as the ones described by Guinney and colleagues for each dataset and selected the reference dataset afterwards, thereby maximising the consistency between the different datasets as described below. We started out by reducing all datasets to a common set of 10,044 reference genes based on the HGNC-symbol-assignments described in the previous sections. Next, each of the reference genes was temporarily represented by the primary identifier (AffyID, Probe ID, etc.) with the largest MAD in each of the datasets (reference identifiers). For each dataset, we then calculated the correlation between all primary identifiers and these reference identifiers, generating ten correlation matrices with a number of rows equal to the number of primary identifiers in the respective dataset and 10,044 columns each; one for each reference identifier. For each dataset, this correlation matrix is equivalent to the collection of correlation vectors C of length $|G_{REF}|$ mentioned in the publication by Guinney and colleagues. For each of the reference genes and each dataset, we then calculated the correlation of all correlation vectors associated with primary identifiers quantifying the respective reference gene to all other correlation vectors from the different datasets associated with primary identifiers quantifying the same reference gene. This resulted in a collection of 10,044 "gene-

lists", one for each reference gene. Each of these "gene-lists" itself consisted of ten "reference-dataset-lists", one for each dataset. Each of these "reference-dataset-lists" itself contained a "dataset-list" of correlation matrices, one for each dataset. These correlation matrices finally contained the correlations of all correlation vectors associated with primary IDs quantifying the respective reference gene in the respective dataset against all corresponding correlation vectors associated with primary IDs quantifying the same reference gene in all other datasets. This enabled us to avoid having to select a reference dataset *a priori* as described by Guinney et al., thereby allowing different selection criteria to be consulted before deciding on a reference dataset. In order to select a reference dataset, we used the following heuristic: For each potential reference dataset and each primary ID in it, we calculated a consistency metric *comet* by first determining which primary IDs in the other datasets quantifying the same reference gene show the highest "correlation of correlations" value with the respective primary ID in the potential reference dataset and subsequently summing up these "correlations of correlations" for each primary ID in the potential reference dataset. For each dataset and reference gene, this resulted in a vector of *comet* values, one for each primary ID quantifying the respective reference gene. The primary ID with the maximum *comet* value (*maxcomet* value) was selected to represent the respective reference gene in the respective dataset. These primary IDs were the ones resulting in the highest consistency between the different datasets if we were to select the respective dataset as a reference. We then compared the distribution of these *maxcomet* values across all datasets in order to find the reference dataset resulting in the highest consistency between the different datasets. Four of the ten datasets showed similarly promising distributions of *maxcomet* values (E-MTAB-2706, GSE28567, GSE8332 and GSE36133) and we decided to use GSE36133 as the reference dataset, since it was the biggest dataset in terms of both primary IDs and cell lines. In this reference dataset, primary IDs associated with *maxcomet* values were selected to represent their corresponding reference genes. For each of the other datasets, the primary ID with the maximum "correlation of correlations" value with the primary ID chosen to represent a given reference gene in the reference dataset was selected to represent the respective reference gene in the respective dataset. This resulted in ten datasets with 10,044 genes each, which were represented by a combination of primary IDs ensuring maximum consistency between the different datasets.

We next merged the eight datasets quantifying transcription in cell lines into one expression matrix and also generated a second expression matrix containing all ten datasets. These two expression matrices were treated separately from one another during adjustment of batch effects in order to maximise the number of genes in each of them. First, we reduced these matrices to genes quantified in at least two samples per dataset, followed

by the adjustment of batch effects between the different datasets using ComBat (Johnson et al, 2007) with MSI status as a covariate. Next, we restricted the expression matrix containing all ten datasets to samples also present in the CPTAC patient dataset. At this stage, we had two mRNA expression matrices, which–together with the giBAQ quantitation for cell lines and patients described above–served as the basis for our multi-omics data integration strategy.

Multi-omics data integration strategy

Computation of protein/mRNA fold-changes

Wilhelm et al. showed that protein/mRNA ratios or fold-changes are reasonably stable across a number of different tissues and that median ratios can be used to estimate protein abundance from mRNA abundance for a given protein (Wilhelm et al, 2014). Since these fold-changes were calculated based on RPKM values as determined by mRNA-Seq, we were curious as to whether this also holds true when transcript abundance is estimated using microarrays. We therefore calculated the median expression for each transcript and sample in the two mRNA expression matrices described above and divided the giBAQ values of the corresponding proteins in the CRC65 cell line and CPTAC patient dataset by these values in a sample-wise manner. This resulted in two ratio matrices, one for the CRC65 dataset and one for the CPTAC dataset, which were used to produce Figures EV3A & B. The median mRNA expression values were the basis for Figure EV2B. These figures showed that there are systematic differences between protein and mRNA datasets, which manifest as reasonably stable protein/mRNA fold-changes within the CRC65 and CPTAC datasets. Notably, the greater proteomic 'depth' of the CRC65 dataset resulted in systematically higher protein/mRNA fold-changes compared to the CPTAC dataset. This could not have been adjusted using global total-sum normalisation, since the peptide coverage differed drastically between the two datasets in a non-linear way (Fig. EV2C).

Integration of transcriptomics and proteomics

Because protein/mRNA fold-changes were reasonably stable within both datasets, we hypothesised that they could be treated as systematic differences and therefore be adjusted using e.g. ComBat as described above. ComBat however shifts genes in each dataset to the overall mean and pooled variance across datasets, thereby altering the location and scale of each dataset. In our case, we wanted to avoid altering the giBAQ expression values as they represent actual measurements of gene group abundance on the protein level. Therefore, we decided to instead use MComBat (Stein et al, 2015), which allows the specification of a "gold-standard" dataset

towards which all other datasets are adjusted and which is not changed. MComBat takes advantage of the systematic differences between protein and mRNA measurements and models them in a protein-wise fashion. Stein and colleagues published the R code for MComBat alongside their manuscript at https://github.com/SteinCK/M-ComBat/blob/master/M-ComBat%20R%20Script, however this script is setup to stop if the combined expression matrix used as input contains any number of missing values. We modified this script to instead work similarly to the original ComBat function implemented in the sva R package v3.18.0 with respect to missing values; the modified script is available at https://github.com/mfrejno/pharmacoproteomics_crc. With this modified MComBat function at hand, we set out to integrate transcriptomics and proteomics data for the CRC65 cell line and CPTAC patient datasets separately from one another. After merging proteomics and transcriptomics data for each dataset separately, we reduced these matrices to gene groups quantified in at least two samples on both the protein and transcript level, followed by the adjustment of systematic differences between proteomics and transcriptomics data using the aforementioned modified MComBat function without MSI status as a covariate. The resulting adjusted expression matrices were the basis for Figure EV3C, as well as for mRNA-guided missing value imputation. Preserving differences between mRNA measurements of distinct cell lines, this adjustment resulted in protein measurements of one cell line to cluster together with mRNA measurements of the same cell line. Before, protein and mRNA measurements clustered together in their respective dataset and were not similar to each other. Here, we would like to stress that we only made use of MComBat in order to be able to perform mRNA-guided missing value imputation. The increase in correlation between protein and mRNA data is only due to the fact that MComBat removed systematic differences between them.

mRNA-guided missing value imputation

The increase in the overall correlation between transcriptomics and proteomics data after adjusting for systematic differences between them (see Fig. EV2B and EV3C) enabled calculating protein abundance from adjusted mRNA abundance. In order to do so, the CRC65 and CPTAC datasets had to be processed separately from one another. For each protein sample and all of its cognate mRNA samples, we assembled all pairs of protein and mRNA measurements corresponding to the same gene group and subsequently modelled protein abundance as a function of adjusted mRNA abundance using a single linear model. For each cognate mRNA sample, we then used the corresponding linear model to calculate the protein abundance of gene groups with missing values in the respective protein sample. For each gene group with missing values in the respective protein sample, we then used the mean across all calculated protein abundances based on cognate mRNA

samples to impute the giBAQ value of the corresponding gene group. Imputed giBAQ values smaller than zero were treated as missing values (see Fig. EV2D).

Minimum-guided missing value imputation

Since we were not able to impute all missing values in the proteomics datasets using mRNA-guided missing value imputation, we explored a number of secondary imputation methods, which could be used after mRNA-guided missing value imputation (Figure EV2E). All of these methods shared the common assumption that most missing values in proteomics datasets are "missing not at random" (MNAR), i.e. that the likelihood of "encountering" a missing value increases as the abundance of the respective analyte approaches the detection limit of the instrument (Lazar et al, 2016). We first confirmed that this assumption holds true for both the CRC65 cell line and CPTAC patient datasets by calculating the frequency and fraction of missing values per intensity bin for both datasets after MComBat adjustment but before mRNA-guided missing value imputation, which was the basis for Figure EV2A. The first imputation method we tried out falls into the MinProb category described by Lazar and colleagues and was the method described in Figure EV3 of the recent publication by Tyanova et al (2016), using a down-shift of 1.8 and a width of 0.3. When we applied this method before mRNA-guided missing value imputation, this resulted in strong bimodality of the intensity distribution for both datasets, but even applying it after mRNA-guided missing value imputation did not circumvent this undesirable behaviour completely. When adjusting the parameters of this perseus-type imputation such that the imputed values became more and more (eventually fully) part of the overall distribution, we found an ever increasing and eventually large number of cases in which the imputed values for specific proteins had much higher intensities than the same but experimentally robustly measured protein intensities in a different sample, which is equally undesirable. Since imputing missing values with the sample-wise (MinDet method described by Lazar et al.) or protein-wise minimum introduced too much bias by reducing the variability of the measurements, we decided to implement a new imputation method termed "minimum-guided missing value imputation" on the protein level. This method ensured that no imputed value for a given protein was bigger than a measured value for the same protein by sampling missing values with replacement from the distribution of measured values smaller than the minimum for the respective protein. This was done across the entire dataset, which is why each dataset needed to be normalised and handled separately. For each protein with one or more missing values across the dataset, we first determined the minimum expression value. In a protein-wise fashion, missing values were subsequently replaced with measured values, which were sampled from the truncated distribution of measured values as described above. This resulted in a much more favourable behaviour with reduced bimodality of the intensity

distributions of each dataset if applied after mRNA-guided missing value imputation. Imputation on the protein level however should not be favoured over imputation on the peptide level, since aggregating peptide-level information to form protein expression values already represents a form of implicit missing value imputation, as discussed by Lazar et al (2016). We therefore decided to transfer the concept of minimum-guided missing value imputation to the peptide level, but only imputed missing values for peptides if the corresponding protein abundance was missing as well. With a stable protein-group-to-HGNC-symbol mapping at hand (see above), we made use of MaxQuant's relational database output and mapped each peptide and its intensity in the evidence.txt output file to the protein group and by extension also to the gene group to the quantification of which the respective peptide intensity contributed. Since razor peptides per definition map to more than one protein group, we made sure to map their intensity only to those protein and gene groups for the quantification of which MaxQuant actually used them. For each protein with one or more missing values across the dataset, we then found the sample with the lowest expression of this protein and determined which peptides contributed to this value. For each missing value in each of these reference peptides, we then searched in a window of 1.1 min (centred at the recalibrated retention time of the respective reference peptide in the matching fractions) for peptides with a lower intensity than the reference peptide. From the intensities of these low-intensity peptides, we then sampled the missing values for the corresponding reference peptide with replacement. All imputed peptide intensities corresponding to a given protein with missing values were subsequently summed up in a sample-wise fashion to form protein intensities and calculate giBAQ values as described above. By adjusting the retention time window queried for low-intensity peptides, one can control how similar the imputed values will be to one another, thereby reducing the variability across the dataset in the low-intensity range. This is useful especially for clustering algorithms using correlation as a measure of distance between samples, since low-intensity features will not contribute as much to the distance between samples as they would have if they were imputed with a different method, giving more weight to reliably measured features. Here, we decided to use the same window MaxQuant used for transferring identifications by "Match between runs", since the concept is somewhat similar. Missing values in our Kinobeads dataset were imputed using minimum-guided missing value imputation on the gene group level. It is worth noting that the clustering results were the same ($p<0.0005$, two-sided Fisher's Exact Test) and the fold-changes of significantly differentially expressed proteins were highly correlated (Pearson's $R=0.997$) when using the perseus-type or minimum-guided missing value imputation.

After this two-step imputation, 93.6/74.7% of all values in the CRC65/CPTAC dataset were measured at the protein level, 2.7/10.9% were measured at the mRNA level and 3.7/14.3% were imputed using minimum-guided

missing value imputation on the peptide level, respectively. The Kinobeads dataset contained 5.3% missing values before imputation as described above.

Combination of the CRC65 and CPTAC datasets

After missing value imputation, the CRC65 cell line and CPTAC patient datasets had to be combined into one expression matrix in order to be able to identify integrated proteomic subtypes of CRC consisting of cell lines and patients. However, the imputation we performed might influence downstream analyses, since some of the proteomics samples had more missing values than others and because we did not have transcriptomics data for all samples. Missing values in samples for which we did not have data on the mRNA level were imputed using minimum-guided missing value imputation only, possibly generating outlier samples, which will distort any unsupervised clustering. We therefore first removed outlier samples from both datasets separately using arrayQualityMetrics, only keeping samples not marked as an outlier by any of the outlier detection methods performed ("Distance between arrays", "Boxplots" and "MA plots"). Afterwards, we merged the two expression matrices into one joined matrix. Since we identified substantially more peptides per sample in the CRC65 dataset than in the CPTAC dataset (Fig. 2 and EV2C) and because MaxQuant calculates protein intensities as the sum of peptide intensities, we expected strong systematic differences between the two datasets, which would have an unfavourable impact on any clustering algorithm applied to the joined expression matrix. Therefore, we adjusted systematic differences between the two datasets using ComBat (Johnson et al, 2007) with MSI status as a covariate as described above.

Identification & characterisation of subtypes

Identification of subtypes

After combining the full proteome datasets into one expression matrix and following the removal of systematic differences between them, we used consensus clustering (Monti et al, 2003) as implemented in the ConsensusClusterPlus R package v1.24.0 to determine Full Proteome Subtypes (FPSs) and Kinobeads Subtypes (KSs) of CRC. The method we used was in many aspects similar to the method used by Zhang et al (2014), but made use of a different agglomeration method, while being more stringent during the identification of core samples. The main difference however was that we used the entire expression matrix for the discovery of FPSs and only restricted our Kinobeads data to kinases during the identification of KSs. We used hierarchical clustering to assign samples to one of k=4 clusters, with "1-Pearson correlation" as the distance metric,

"ward.D2" as the agglomeration method and 1,000 resampling iterations using 80% of all gene groups and samples, while varying k from two to eight clusters during exploratory data analysis. We decided to use four clusters based on the four known Consensus Molecular Subtypes published by Guinney et al (2015). Clusters with less than six members were removed, followed by the calculation of item-consensus values and silhouette widths (Rousseeuw, 1987) for each sample using the calcICL function from the ConsenusClusterPlus R package v1.24.0 and the silhouette function of the R package cluster v2.0.3, respectively. Being more stringent than Zhang et al., we defined core samples as samples with a positive silhouette width, provided that they also show the highest item-consensus with the cluster they were finally assigned to. Clusters only consisting of core samples for which we did not have mRNA data were excluded in order to avoid artefacts due to missing value imputation. The columns in the final heat maps were restricted to core samples, which were ordered according to the final consensus tree. This method was also used to cluster the reduced mRNA expression matrix after the exclusion of cell lines not in the CRC65 dataset and transcripts not in the CMS classifier by Guinney et al. (2015), in order to produce Figure 1B.

Differential expression analysis

We used significance analysis of microarrays (SAM; Tusher et al, 2001) as implemented in the samr R package v2.0 in order to discover gene groups differentially expressed between the different subtypes we identified, restricting the analysis to core samples while using 100 permutations, the Wilcoxon test statistic and a target FDR of 0.01 (1%). The rows in the final heat maps were ordered according to a similar metric, which was intended to visualise which gene groups were higher or lower expressed in one subtype compared to the remaining subtypes. First, we standardised the expression matrix in a row-wise fashion so that each gene group had a mean of zero and a standard deviation of one. For each gene group, we then calculated all pairwise combinations of two-sided Wilcoxon test statistics between each subtype and the remaining subtypes. Each gene group was then assigned to the subtype with the lowest p-value. For each subtype, gene groups associated with it were then ordered in increasing order of their summed expression across all samples in this subtype, starting with the gene groups with higher expression in the respective subtype compared to the others, followed by gene groups with lower expression in the respective subtype compared to the others. These blocks of gene groups were then sorted according to the order of the subtypes they were associated with in the final consensus tree, creating the checkerboard-like pattern seen in Figures 3A and Figure EV5G.

Functional annotation enrichment & clustering

We used MetaCore v6.26.68498 from Thomson Reuters to perform enrichment analyses of functional annotations for the gene groups differentially expressed between the different FPSs. In order to be more stringent, we set the background MetaCore uses to calculate its p-values for the enrichment of functional annotations using the hypergeometric test to the list of network objects defined by the HGNC symbols detected in at least two samples in both the CRC65 cell line and CPTAC patient datasets. This ensures that the enrichment analysis does not suffer from acquisition bias. The standardized mean difference between subtype-specific gene group expression and overall mean expression as computed using SAM served as the raw input for the enrichment analysis, which was further filtered to remove values between the 2.5% and 97.5% quantiles of these values across all permutations, in order to restrict the analysis to gene groups with large class contrasts. This filtered matrix was uploaded to https://portal.genego.com/, followed by the calculation of enrichment analyses with respect to “Pathway Maps”, “Process Networks”, “GO Processes”, “GO Molecular Functions” and “GO Localizations” for up- and down-regulated gene groups, respectively. We exported the entire table of annotations for each of these functional annotation enrichments and further processed them in R. For “Pathway Maps” and “Process Networks”, we only imported annotations with a minimum FDR≤0.05 (5%) as provided by MetaCore, merged the tables of annotations enriched in up- and down-regulated gene groups from any subtype into a single table, excluded rodent-specific annotations and subsequently assembled bar charts of $-\log_{10}$(FDR) values for up- and down-regulated gene groups into a single figure. For each of the different Gene Ontology (GO) categories, we also only imported annotations with a minimum FDR≤0.05 (5%) as provided by MetaCore. Subsequently, tables for the different GO categories were combined while keeping GO-terms enriched in up- and down-regulated gene groups specific to a certain subtype separate from one another. This resulted in six different tables; two for each subtype containing GO-terms associated with up- and down-regulated gene groups, respectively. Afterwards, GO-term names were re-mapped to GO Accessions using a mapping downloaded from AmiGO using GOOSE (http://amigo.geneontology.org/goose, 19 May 2016) and GO Accessions with an FDR of more than 0.05 (5%) were removed from the corresponding table. Finally, these tables were sequentially uploaded to REViGO (http://revigo.irb.hr/) for summarisation and visualisation of significant GO-terms using treemaps, with an allowed similarity of 0.7, interpreting associated numbers as p-values, restricting the database to Homo sapiens and employing the SimRel semantic similarity measure (01 July 2016; Schlicker et al, 2006).

Reanalysis of public dose-response datasets

Pre-processing

The same cell lines and drugs have inconsistent names in the different drug sensitivity datasets, which is why the first pre-processing step always involved the mapping of all cell line and drug names to a set of consistent sample and drug names, respectively (see also Tables EV6A and 6B). For reasons of transparency, we also annotated each drug with the corresponding dataset it was derived from. Concordant cell lines were treated as replicates during model fitting, which was kept as consistent as possible between the different datasets. In essence, we always modelled a relative response measure R as a function of the final drug concentration on the assay plate as described below.

GDSC

We obtained raw dose-response data (gdsc_drug_sensitivity_raw_data_w5.csv), annotation information (gdsc_tissue_output_w5.csv) and screening concentrations (gdsc_compounds_conc_w5.csv) from ftp://ftp.sanger.ac.uk/pub4/cancerrxgene/releases/release-5.0/ (04 September 2015) prior to the publication by Iorio et al (2016), which is why all our analyses were based on release v5.0. First, we annotated the raw data with the corresponding drug names and "fold-dilution" information, as well as data on the maximum screening concentration used for each drug. This allowed us to calculate the actual concentrations at which each compound was used in the drug screen. We then calculated the mean background signal for each compound and cell line (blank wells not containing cells) and subtracted it from each corresponding raw signal, setting negative values to zero afterwards. Next, we determined the mean control signal for each compound and cell line and afterwards divided each corresponding background-corrected raw signal by its respective control signal, generating normalised and background-corrected viability data. Finally, for each compound, compound concentration and cell line, we then calculated relative response measures R as the mean of these values and restricted the dataset to cell lines from the large intestine, as well as C32 and SW626.

CCLE

We downloaded Supplementary Table 11 from the original publication by Barretina et al (2012) and restricted the dataset first to cell lines from the large intestine, as well as C32 and Colo 741. We then restricted the dataset further to only include drugs for which the authors were able to fit at least two sigmoid dose-response models among our selection of cell lines as indicated in the FitType column, removing dose-response data for both "Nutlin-3" and "PHA-665752". Subsequently, we converted raw activity values A ("Activity Data (median)")

back to relative response measures R=T/U=1+A/100 (T represents the response for the compound-treated well, U represents the median response of the untreated wells across the plate) by reverting the corresponding equation in the addendum to the original publication, in order to make the CCLE dataset more comparable to the other drug sensitivity datasets.

CTRP

We obtained the expanded CTRP v2.0 dataset from the FTP server of the CTD^2 data portal at ftp://caftpd.nci.nih.gov/pub/dcc_ctd2/Broad/CTRPv2.0_2015_ctd2_ExpandedDataset/ (23 December 2015). The results published here are therefore partially based upon data generated by the Cancer Target Discovery and Development (CTD2) Network (https://ctd2.nci.nih.gov/dataPortal/) established by the National Cancer Institute's Office of Cancer Genomics. First, we annotated the raw data with the corresponding compound and cell line names, as well as information on the tissue of origin for the cell lines used in the screen. Based on these annotations, the dataset was restricted to cell lines from the large intestine, as well as C32 and Colo 741. Viability data annotated with the same cell line name, compound concentration and compound name were treated as replicates during model fitting. The weighted percent-viability with error propagation ("cpd_avg_pv") was used as a relative response measure R.

Cetuximab

We downloaded (03 January 2016) Supplementary Data 1 from the original publication by Medico et al (2015) and converted the percentage of growth inhibition P to a relative response measure R=1-P/100 in order to make the data comparable to the other drug sensitivity datasets.

Dose-response models & parameter extraction

Using the drc package v2.5-12 in R, we fitted the classical symmetric four-parameter log-logistic model to each drug in each dataset:

$$f(x,(b,c,d,e)) = c + \frac{d-c}{1+\exp\left(b(\log(x)-\log(e))\right)}$$

The four parameters c="Lower Limit" (i.e. lower limit of the relative response as the drug concentration approaches infinity), d="Upper Limit" (i.e. upper limit of the relative response as the drug concentration approaches zero), b="Slope" (Hill slope, i.e. slope at the inflection point of the dose-response curve), and e="ED50" (i.e. the dose required to reduce the relative response to c+(d-c)/2) were estimated from the data and subsequently extracted from the fitted model. We also modified the computeAUC function from the drexplorer

package v1.1.2 to accept the output of the drm function from the drc R package as an input and used it to extract the standardised area under the dose-response curve (AUC) for each of the fitted models across the tested dose-range. Here, the AUC across the tested dose-range was defined as the area under the dose-response curve between zero and one, divided by the area under y=1 from the lowest to the highest concentration tested. It is worth noting that the relative response measures described above varied between approximately zero and one, enabling unified model fitting and parameter extraction.

Modelling of Drug Sensitivity

In order to find proteins and kinases associated with drug sensitivity or resistance, we used elastic net regression (Zou & Hastie, 2005) to model drug sensitivity as a function of protein and kinase profile, respectively. Together with a bootstrapping approach, this regularised multivariate linear regression method was previously successfully applied to identify genomic, transcriptomic and proteomic markers of drug sensitivity and resistance (Barretina et al, 2012; Garnett et al, 2012; Gholami et al, 2013; Iorio et al, 2016). The advantages of the elastic net are discussed in detail in the Supplementary Methods associated with the publication by Barretina et al (2012), as well as in the original publication by Zou and Hastie (2005). Briefly, the algorithm is especially useful for "large p, small N" ($p>>N$) regression problems involving for example data generated using current omics technologies of various kind, which measure many, possibly highly correlated analytes (p) across a small number of samples (N). In settings like these with varying degrees of multicollinearity, the ordinary least squares (OLS) estimates of the regression coefficients and the resulting models frequently perform poorly both in terms of prediction accuracy on new data, as well as regarding the interpretability of the model itself (Zou & Hastie, 2005). The elastic net aims at improving both prediction performance and model interpretability by consolidating L2-regularised Ridge regression (Hoerl & Kennard, 1970) and L1-regularised Lasso regression (Tibshirani, 1996), in order to combine the advantages of the different penalty terms they use to constrain the coefficient vector. On its own, Ridge regression often has a higher predictive accuracy than OLS in the above-mentioned situations by striking a balance between bias and variance of predicted values via its bound on the L2-norm of the coefficient vector, thereby aiming at improving the overall prediction accuracy (Tibshirani, 1996). However, it always keeps all predictors in the model, which is not useful if the goal is to increase its interpretability compared to OLS. Lasso regression on the other hand does automatic variable selection via its bound on the L1-norm of the coefficient vector, thereby shrinking some coefficients while forcing others to be exactly zero, which results in a more parsimonious model consisting of a reduced subset of good predictors,

which is easier to interpret (Tibshirani, 1996). However, this comes at the cost of only selecting at most N variables in settings where p>>N, as well as only selecting one variable from a group of correlated variables to be included in the model while excluding the others in settings with multicollinearity (Zou & Hastie, 2005). The authors also observed that Ridge regression performs far better than Lasso regression with respect to predictions on new data in settings with multicollinearity where N>p. By combining these two penalty terms, the elastic net now promotes parsimony of models through the L1-penalty, while at the same time encouraging a grouping effect through the L2-penalty (Barretina et al, 2012), meaning that highly collinear predictors are usually either in the model or dropped from it together. Double shrinkage of coefficients is prevented by the introduction of a scaling factor (Zou & Hastie, 2005) and the higher level parameter α with $0 \leq \alpha \leq 1$ is used to control the balance between the L2-penalty (α=0) and the L1-penalty (α=1), while λ controls the degree of regularisation (Friedman et al, 2010).

If $y_i \in \mathbb{R}^N$ are drug responses expressed as AUC for i=1,…,N cell lines and $x_i \in \mathbb{R}^N$ are standardised measurements for i=1,…,N cell lines across j=1,…,p proteins or kinases with zero mean and unit variance forming N observation pairs (x_i, y_i), then the cost function we minimised using glmnet v2.0-5 (Friedman et al, 2010) is given by

$$\min_{(\beta_0,\beta)\in\mathbb{R}^{p+1}} \frac{1}{2N}\sum_{i=1}^{N}(y_i - \beta_0 - x_i{}^T\beta)^2 + \lambda\left[(1-\alpha)\frac{1}{2}\|\beta\|_{l_2}^2 + \alpha\|\beta\|_{l_1}\right]$$

We used leave-one-out cross-validation over a grid of 100 equally-spaced values of α with $0 \leq \alpha \leq 1$ by 100 values of λ automatically selected by the algorithm for each α to find the combination of α and λ resulting in the lowest mean-squared error between the fitted mean and the response. We then turned to a bootstrapping approach similar to the ones used before in order to be able to sort the different proteins or kinases according to their importance in predicting the response to a given drug (Barretina et al, 2012; Garnett et al, 2012; Gholami et al, 2013; Iorio et al, 2016). Briefly, we generated 1000 bootstrap datasets by sampling the above-mentioned observation pairs with replacement and solved the elastic net optimisation problem for each of these datasets using the optimal values of α and λ. This resulted in a $1000 \times (p+1)$ matrix of regression coefficients capturing the solutions for the bootstrap datasets as rows and the regression coefficients as columns. The intercept column $\beta_{int}^{BS} \in \mathbb{R}^{1000,1}$ was stored separately from the remainder of the matrix, which left us with a $1000 \times p$ matrix of coefficients $\beta_{coef}^{BS} \in \mathbb{R}^{1000,p}$ for j=1,…,p proteins or kinases across 1000 bootstrap datasets. Each column of β_{coef}^{BS} was subsequently multiplied by the standard deviation of the corresponding protein or kinase across all x_i, as well as by its corresponding weight ω described in Equation 11 in the publication by

Moghaddas Gholami et al (2013) if $\omega \in \{-1,1\}$ or by zero if $\omega \notin \{-1,1\}$. This resulted in a $1000 \times p$ matrix of effect sizes β_{eff}^{BS} analogous to Equation 12 in the publication by Moghaddas Gholami et al. It is worth stressing that columns of effect sizes corresponding to proteins or kinases whose coefficients did not have a consistent sign across all 1000 bootstrap solutions were set to zero through the multiplication with the weight ω. For each column in β_{int}^{BS}, β_{coef}^{BS} and β_{eff}^{BS}, we then calculated the mean across all bootstrap solutions to form β_{int}^{mean}, β_{coef}^{mean} and β_{eff}^{mean}, respectively, and also counted the number of times the absolute effect size of a given protein or kinase was bigger than zero to form $F_{eff} \in \mathbb{N}^p$. For each protein or kinase j=1,…,p, we then determined whether (a) $F_{eff,j}$ was bigger than or equal to the 9th decile of F_{eff} and also checked whether $\beta_{eff,j}^{mean}$ was either (b_1) bigger than or equal to the 19th ventile of β_{eff}^{mean} or (b_2) smaller than or equal to the 1st ventile of β_{eff}^{mean}. We only considered proteins or kinases for which both condition (a) and one of condition (b_1) or (b_2) were true to be robust predictive markers of drug response, while all proteins or kinases j=1,…,p with $\left|\beta_{eff,j}^{mean}\right| > 0$ were designated as being associated with drug response. In effect-size heat maps, we only displayed the top 5 and bottom 5 robust predictive markers of drug response with respect to β_{eff}^{mean}, while restricting the columns to only the top 10 and bottom 10 cell lines with respect to their drug response as measured by AUC. For each drug, we always used all N available observation pairs (x_i, y_i) to train the model with three different input matrices each:

1) The combined full proteome expression matrix after missing value imputation
2) All proteins in the Kinobeads expression matrix after missing value imputation
3) All kinases in the Kinobeads expression matrix after missing value imputation

For each drug, we then used the corresponding β_{int}^{mean} and β_{coef}^{mean} from scenario 1) to predict the drug response $\hat{y}_i$ of each cell line and patient in the combined dataset based on the corresponding full proteome expression profile x_i using the following formula:

$$\hat{y}_i = \beta_{int}^{mean} + \sum_{j=1}^{p} \beta_{coef,j}^{mean} \times x_{i,j}$$

These predictions were subsequently standardised to have zero mean and unit variance and can be found in Table EV3A. We also predicted $\hat{y}_i$ using β_{int}^{mean} and β_{coef}^{mean} from scenario 3) and subsequently standardised these predictions to have zero mean and unit variance in order to generate kinase-centric drug sensitivity hypotheses. Cell lines with a standardised AUC bigger than zero were designated as “resistant”, while cell lines with a standardised AUC smaller than zero were designated as “sensitive”. The type of figure used to visualise

the result of an elastic net regression is termed "effect-size heat map". In effect-size heat maps, up to five of the most robust predictive markers of drug resistance and sensitivity with respect to β_{eff}^{mean} were displayed, respectively, while restricting the columns to at most the top 10 and bottom 10 cell lines with respect to their drug response as measured by AUC in order to increase visual clarity. In such a figure, these top and bottom ten cell lines are shown as columns ordered from left to right in increasing order of drug resistance, while the predictors (proteins or kinases) with respect to absolute effect-size are shown as rows. In the bar plot to the left, predictors associated with drug resistance are visualised by yellow bars, while predictors associated with drug sensitivity are shown as dark blue bars in increasing or decreasing order of absolute effect-size from top to bottom, respectively. The heat map itself shows standardized expression values (z-scores) of the predictors with blue="low expression" and red="high expression". Below the heat map, a heat strip visualises the AUC of the drug in question from dark-blue="low AUC" to yellow="high AUC" (see Figure 1A for an example of an effect-size heat map).

Cox proportional hazards regression

In order to assess whether or not the cytoplasmic/membranous expression level of MERTK is a prognostic biomarker in CRC, we modelled the different outcome variables of the QUASAR 2 trial as a function of MERTK expression using univariate and multivariate Cox proportional hazards regression (COXPH), restricted to the first 5 years of follow-up. First, univariate COXPH was carried out using the coxph function of the R package survival v2.38-3 with default parameters and "Gender", "Age", "BMI", "Treatment" (capecitabine ± bevacizumab), "Location" (right colon or left colon), "T" (from TNM staging system; T2/T3 or T4), "N" (from TNM staging system; N0, N1 or N2), "MSI" (microsatellite instability), "CIN" (chromosomal instability) and "MERTK" (cytoplasmic/membranous MERTK expression; ≤5% or >5%) as covariates. The threshold of 5% was selected in order to balance the groups while avoiding false positives due to weak staining. Significant ($p<0.05$) covariates from univariate COXPH were then used as explanatory variables in multivariate COXPH. We made use of the stepAIC function from the R package MASS v7.3-45 to perform stepwise backward model selection starting with the full model and removing explanatory variables in order to minimise Akaike's Information Criterion (AIC). For each outcome variable, final models were then built using significant ($p<0.05$) covariates from the stepwise selected models. These final models all fulfilled the proportional hazards assumption. Results from the COXPH analyses can be found in Table EV5.

Annotation information

We compiled annotation information for the CRC65 and CPTAC datasets, as well as target annotations for drugs in the four drug sensitivity datasets (GDSC, CCLE, CTRP and Cetuximab) from several sources (Supplementary Table 11 & GSE36133, Barretina et al., 2012; Supplementary Table 1, Bracht et al., 2010; Supplementary Table 3, De Sousa et al., 2013; COSMIC, Forbes et al., 2015; GDSC release 5.0 & E-MTAB-783, Garnett et al., 2012; cms_labels_public_all.txt, Guinney et al., 2015; GDSC release 5.0, Iorio et al., 2016; Figure 1 & E-MTAB-2706, Klijn et al., 2015; Table 1, Ku et al., 1999; Table S1, Liu and Zhang, 2016; GSE28567, Loboda et al., 2011; Supplementary Data 1 & 2 & GSE59857, Medico et al., 2015; Supplementary Table 1 & 6, Mouradov et al., 2014; CTRP v2.0 expanded dataset, Rees et al., 2016; Supplementary Table 2, Sadanandam et al., 2013; Supplementary Table 1 & platform codes "IlluminaGA_RNASeq" & "AgilentG4502A_07_3", TCGA Network, 2012; GSE8332, Wagner et al., 2007; Table 1, Wheeler et al., 1999; GSE24795, Wilding et al., 2010; Supplementary Table 1, Zhang et al., 2014) and summarised them in Table EV6. Entries for cell lines in the columns "Sadanandam subtype", "Marisa subtype", "De Sousa E Melo subtype" and "Roepman subtype" were based on the publication by Medico and colleagues and were not restricted by an FDR filter. The "Goblet-like", "Goblet" and "Enterocyte" subtype of the classifier by Sadanandam and colleagues were merged into the "Entererocyte/Goblet-like" subtype, while "MSI-L" was recoded to "MSI-" and "MSI-H" was recoded to "MSI+" according to the publication by Liu and Zhang. Some of the other annotations were also recoded to fit the unified nomenclature. For drug targets, we applied the following heuristic: After mapping compounds to a consensus set of names, the annotation from the CTRP dataset was used as the first source of target information. Drugs for which this dataset lacked annotation were assigned targets based on the annotation from the GDSC dataset. Drugs the target information of which was not included in the GDSC dataset were then assigned targets based on the annotation from the CCLE dataset. Drugs still left without target information were finally assigned labels based on the information contained in the "target_or_activity_of_compound" column of the "v20.meta.per_compound.txt" file from the expanded CTRP v2.0 dataset. Amendments to these mappings in order to reduce redundancy are documented in Table EV6B.

Appendix Supplementary References

Barretina J, Caponigro G, Stransky N, Venkatesan K, Margolin AA, Kim S, Wilson CJ, Lehar J, Kryukov GV, Sonkin D, Reddy A, Liu M, Murray L, Berger MF, Monahan JE, Morais P, Meltzer J, Korejwa A, Jane-Valbuena J, Mapa FA et al (2012) The Cancer Cell Line Encyclopedia enables predictive modelling of anticancer drug sensitivity. *Nature* **483:** 603-607

Bracht K, Nicholls AM, Liu Y, Bodmer WF (2010) 5-Fluorouracil response in a large panel of colorectal cancer cell lines is associated with mismatch repair deficiency. *Br J Cancer* **103:** 340-346

De Sousa EMF, Wang X, Jansen M, Fessler E, Trinh A, de Rooij LP, de Jong JH, de Boer OJ, van Leersum R, Bijlsma MF, Rodermond H, van der Heijden M, van Noesel CJ, Tuynman JB, Dekker E, Markowetz F, Medema JP, Vermeulen L (2013) Poor-prognosis colon cancer is defined by a molecularly distinct subtype and develops from serrated precursor lesions. *Nat Med* **19:** 614-618

Edgar R, Domrachev M, Lash AE (2002) Gene Expression Omnibus: NCBI gene expression and hybridization array data repository. *Nucleic Acids Res* **30:** 207-210

Edwards D (2003) Non-linear normalization and background correction in one-channel cDNA microarray studies. *Bioinformatics* **19:** 825-833

Forbes SA, Beare D, Gunasekaran P, Leung K, Bindal N, Boutselakis H, Ding M, Bamford S, Cole C, Ward S, Kok CY, Jia M, De T, Teague JW, Stratton MR, McDermott U, Campbell PJ (2015) COSMIC: exploring the world's knowledge of somatic mutations in human cancer. *Nucleic Acids Res* **43:** D805-811

Friedman J, Hastie T, Tibshirani R (2010) Regularization Paths for Generalized Linear Models via Coordinate Descent. *J Stat Softw* **33:** 1-22

Garnett MJ, Edelman EJ, Heidorn SJ, Greenman CD, Dastur A, Lau KW, Greninger P, Thompson IR, Luo X, Soares J, Liu Q, Iorio F, Surdez D, Chen L, Milano RJ, Bignell GR, Tam AT, Davies H, Stevenson JA, Barthorpe S et al (2012) Systematic identification of genomic markers of drug sensitivity in cancer cells. *Nature* **483:** 570-575

Gholami AM, Hahne H, Wu Z, Auer FJ, Meng C, Wilhelm M, Kuster B (2013) Global proteome analysis of the NCI-60 cell line panel. *Cell Rep* **4:** 609-620

Guinney J, Dienstmann R, Wang X, de Reynies A, Schlicker A, Soneson C, Marisa L, Roepman P, Nyamundanda G, Angelino P, Bot BM, Morris JS, Simon IM, Gerster S, Fessler E, De Sousa EMF, Missiaglia E, Ramay H, Barras D, Homicsko K et al (2015) The consensus molecular subtypes of colorectal cancer. *Nat Med* **21:** 1350-1356

Hoerl AE, Kennard RW (1970) Ridge Regression - Biased Estimation for Nonorthogonal Problems. *Technometrics* **12:** 55-&

Iorio F, Knijnenburg TA, Vis DJ, Bignell GR, Menden MP, Schubert M, Aben N, Goncalves E, Barthorpe S, Lightfoot H, Cokelaer T, Greninger P, van Dyk E, Chang H, de Silva H, Heyn H, Deng X, Egan RK, Liu Q, Mironenko T et al (2016) A Landscape of Pharmacogenomic Interactions in Cancer. *Cell* **166:** 740-754

Johnson WE, Li C, Rabinovic A (2007) Adjusting batch effects in microarray expression data using empirical Bayes methods. *Biostatistics* **8:** 118-127

Klijn C, Durinck S, Stawiski EW, Haverty PM, Jiang Z, Liu H, Degenhardt J, Mayba O, Gnad F, Liu J, Pau G, Reeder J, Cao Y, Mukhyala K, Selvaraj SK, Yu M, Zynda GJ, Brauer MJ, Wu TD, Gentleman RC et al (2015) A comprehensive transcriptional portrait of human cancer cell lines. *Nat Biotechnol* **33:** 306-312

Ku JL, Yoon KA, Kim DY, Park JG (1999) Mutations in hMSH6 alone are not sufficient to cause the microsatellite instability in colorectal cancer cell lines. *European Journal of Cancer* **35:** 1724-1729

Lazar C, Gatto L, Ferro M, Bruley C, Burger T (2016) Accounting for the Multiple Natures of Missing Values in Label-Free Quantitative Proteomics Data Sets to Compare Imputation Strategies. *J Proteome Res* **15:** 1116-1125

Liu Q, Zhang B (2016) Integrative Omics Analysis Reveals Post-Transcriptionally Enhanced Protective Host Response in Colorectal Cancers with Microsatellite Instability. *J Proteome Res* **15:** 766-776

Loboda A, Nebozhyn MV, Watters JW, Buser CA, Shaw PM, Huang PS, Van't Veer L, Tollenaar RA, Jackson DB, Agrawal D, Dai H, Yeatman TJ (2011) EMT is the dominant program in human colon cancer. *BMC Med Genomics* **4:** 9

Medico E, Russo M, Picco G, Cancelliere C, Valtorta E, Corti G, Buscarino M, Isella C, Lamba S, Martinoglio B, Veronese S, Siena S, Sartore-Bianchi A, Beccuti M, Mottolese M, Linnebacher M, Cordero F, Di Nicolantonio F, Bardelli A (2015) The molecular landscape of colorectal cancer cell lines unveils clinically actionable kinase targets. *Nat Commun* **6:** 7002

Monti S, Tamayo P, Mesirov J, Golub T (2003) Consensus clustering: A resampling-based method for class discovery and visualization of gene expression microarray data. *Mach Learn* **52:** 91-118

Mouradov D, Sloggett C, Jorissen RN, Love CG, Li S, Burgess AW, Arango D, Strausberg RL, Buchanan D, Wormald S, O'Connor L, Wilding JL, Bicknell D, Tomlinson IP, Bodmer WF, Mariadason JM, Sieber OM (2014) Colorectal cancer cell lines are representative models of the main molecular subtypes of primary cancer. *Cancer Res* **74:** 3238-3247

Rees MG, Seashore-Ludlow B, Cheah JH, Adams DJ, Price EV, Gill S, Javaid S, Coletti ME, Jones VL, Bodycombe NE, Soule CK, Alexander B, Li A, Montgomery P, Kotz JD, Hon CS, Munoz B, Liefeld T, Dancik V, Haber DA et al (2016) Correlating chemical sensitivity and basal gene expression reveals mechanism of action. *Nat Chem Biol* **12:** 109-116

Ritchie ME, Silver J, Oshlack A, Holmes M, Diyagama D, Holloway A, Smyth GK (2007) A comparison of background correction methods for two-colour microarrays. *Bioinformatics* **23:** 2700-2707

Rousseeuw PJ (1987) Silhouettes - a Graphical Aid to the Interpretation and Validation of Cluster-Analysis. *Journal of Computational and Applied Mathematics* **20:** 53-65

Sadanandam A, Lyssiotis CA, Homicsko K, Collisson EA, Gibb WJ, Wullschleger S, Ostos LC, Lannon WA, Grotzinger C, Del Rio M, Lhermitte B, Olshen AB, Wiedenmann B, Cantley LC, Gray JW, Hanahan D (2013) A colorectal cancer classification system that associates cellular phenotype and responses to therapy. *Nat Med* **19:** 619-625

Schlicker A, Domingues FS, Rahnenfuhrer J, Lengauer T (2006) A new measure for functional similarity of gene products based on Gene Ontology. *BMC Bioinformatics* **7:** 302

Schwanhausser B, Busse D, Li N, Dittmar G, Schuchhardt J, Wolf J, Chen W, Selbach M (2011) Global quantification of mammalian gene expression control. *Nature* **473:** 337-342

Shi W, Oshlack A, Smyth GK (2010) Optimizing the noise versus bias trade-off for Illumina whole genome expression BeadChips. *Nucleic Acids Res* **38:** e204

Silver JD, Ritchie ME, Smyth GK (2009) Microarray background correction: maximum likelihood estimation for the normal-exponential convolution. *Biostatistics* **10:** 352-363

Stein CK, Qu P, Epstein J, Buros A, Rosenthal A, Crowley J, Morgan G, Barlogie B (2015) Removing batch effects from purified plasma cell gene expression microarrays with modified ComBat. *BMC Bioinformatics* **16:** 63

TCGA Network (2011) Integrated genomic analyses of ovarian carcinoma. *Nature* **474:** 609-615

TCGA Network (2012) Comprehensive molecular characterization of human colon and rectal cancer. *Nature* **487:** 330-337

Tibshirani R (1996) Regression shrinkage and selection via the Lasso. *J Roy Stat Soc B Met* **58:** 267-288

Tusher VG, Tibshirani R, Chu G (2001) Significance analysis of microarrays applied to the ionizing radiation response. *Proc Natl Acad Sci U S A* **98:** 5116-5121

Tyanova S, Temu T, Sinitcyn P, Carlson A, Hein MY, Geiger T, Mann M, Cox J (2016) The Perseus computational platform for comprehensive analysis of (prote)omics data. *Nat Methods* **13:** 731-740

Wagner KW, Punnoose EA, Januario T, Lawrence DA, Pitti RM, Lancaster K, Lee D, von Goetz M, Yee SF, Totpal K, Huw L, Katta V, Cavet G, Hymowitz SG, Amler L, Ashkenazi A (2007) Death-receptor O-glycosylation controls tumor-cell sensitivity to the proapoptotic ligand Apo2L/TRAIL. *Nat Med* **13:** 1070-1077

Wheeler JM, Beck NE, Kim HC, Tomlinson IP, Mortensen NJ, Bodmer WF (1999) Mechanisms of inactivation of mismatch repair genes in human colorectal cancer cell lines: the predominant role of hMLH1. *PNAS* **96:** 10296-10301

Wilding JL, McGowan S, Liu Y, Bodmer WF (2010) Replication error deficient and proficient colorectal cancer gene expression differences caused by 3'UTR polyT sequence deletions. *Proc Natl Acad Sci U S A* **107:** 21058-21063

Wilhelm M, Schlegl J, Hahne H, Gholami AM, Lieberenz M, Savitski MM, Ziegler E, Butzmann L, Gessulat S, Marx H, Mathieson T, Lemeer S, Schnatbaum K, Reimer U, Wenschuh H, Mollenhauer M, Slotta-Huspenina J, Boese JH, Bantscheff M, Gerstmair A et al (2014) Mass-spectrometry-based draft of the human proteome. *Nature* **509:** 582-587

Zhang B, Wang J, Wang X, Zhu J, Liu Q, Shi Z, Chambers MC, Zimmerman LJ, Shaddox KF, Kim S, Davies SR, Wang S, Wang P, Kinsinger CR, Rivers RC, Rodriguez H, Townsend RR, Ellis MJ, Carr SA, Tabb DL et al (2014) Proteogenomic characterization of human colon and rectal cancer. *Nature* **513:** 382-387

Zou H, Hastie T (2005) Regularization and variable selection via the elastic net. *Journal of the Royal Statistical Society: Series B (Statistical Methodology)* **67:** 301-320
