# Supplementary material for: Pharmacoproteomic characterisation of human colon and rectal cancer
Source: Mol Syst Biol. 2017 Nov 3;13(11):951. doi: 10.15252/msb.20177701 (PMC5731344; doi:10.15252/msb.20177701)
Supplement: Supplementary file 2 — Expanded View Figures PDF [file MSB-13-951-s002.pdf]

Expanded View Figures

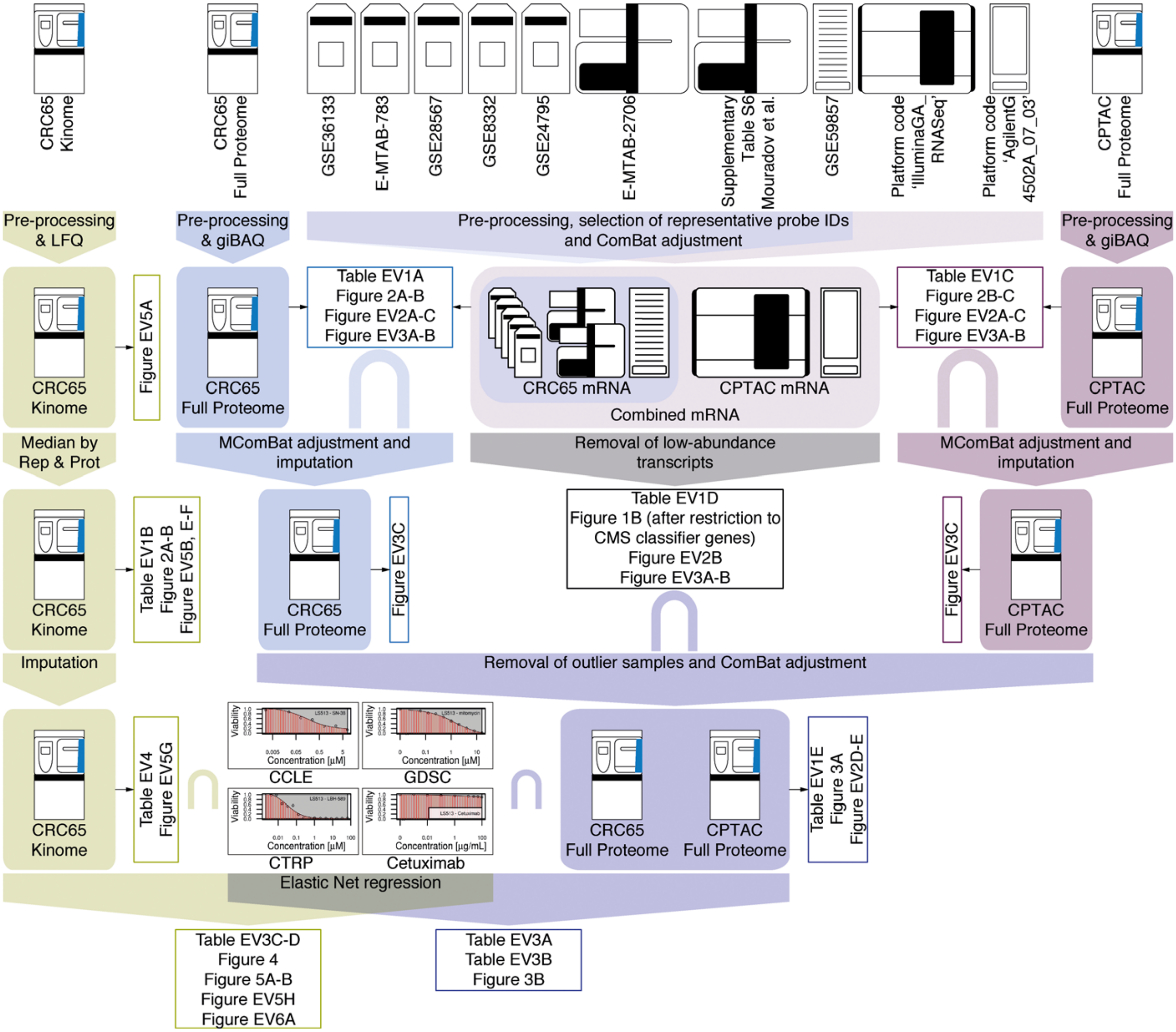

Figure EV1. Data integration pipeline (related to Fig 1).

Overview of the data integration pipeline. Raw data (no box) at the top were subjected to different processing steps (filled box-arrows), which resulted in processed datasets (filled boxes). These were in turn used to generate figures and tables (open boxes). The intersect symbol ∩ was used to denote datasets, which were integrated based on their intersection. The different proteomic datasets were colour-coded as in the main manuscript (green = Kinobeads, blue = CRC65 full proteomes and purple = CPTAC full proteomes; see main text and Appendix Supplementary Methods for details).

© 2017 The Authors

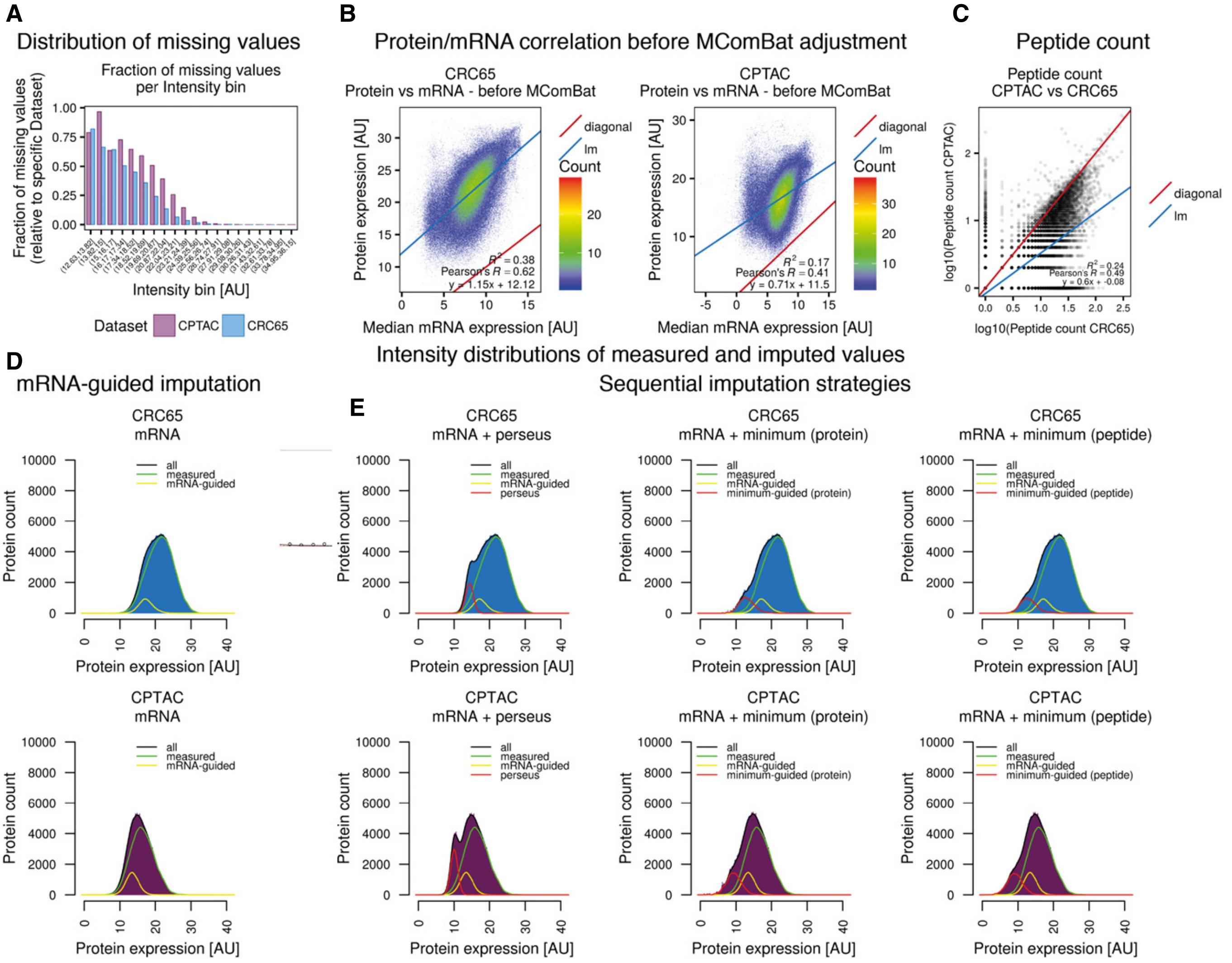

Figure EV2. Protein/mRNA correlation & missing value imputation (related to Fig 3).

A Bar chart of the fraction of missing values per intensity bin, relative to the respective dataset.

B Scatterplot of median mRNA expression before MComBat adjustment for all transcripts across all cell lines versus protein expression in both the CRC65 cell line and CPTAC patient datasets (log2-transformed & median-centred giBAQ; diagonal: $x = y$; lm: linear model; R^2: coefficient of determination).

C Scatterplot of the number of peptides per protein used for the calculation of giBAQ values in the CPTAC dataset versus the CRC65 dataset. The quantification of the majority of all proteins is based on more peptides in the CRC65 dataset compared to the CPTAC dataset (lm: linear model; R^2: coefficient of determination; see main text and Appendix Supplementary Methods for details).

D, E Histograms visualising the distribution of log2-transformed and median-centred giBAQ values of the CRC65 (top row) and CPTAC (bottom row) datasets after application of (D) mRNA-guided, (E) sequential mRNA-guided and perseus-type, as well as sequential mRNA-guided and minimum-guided missing value imputation on the protein or peptide level, respectively. Additional lines visualise the contribution of measured values (green), values imputed by mRNA-guided missing value imputation (yellow) and values imputed by either perseus-type or minimum-guided missing value imputation (red, see also Appendix Supplementary Methods).

© 2017 The Authors

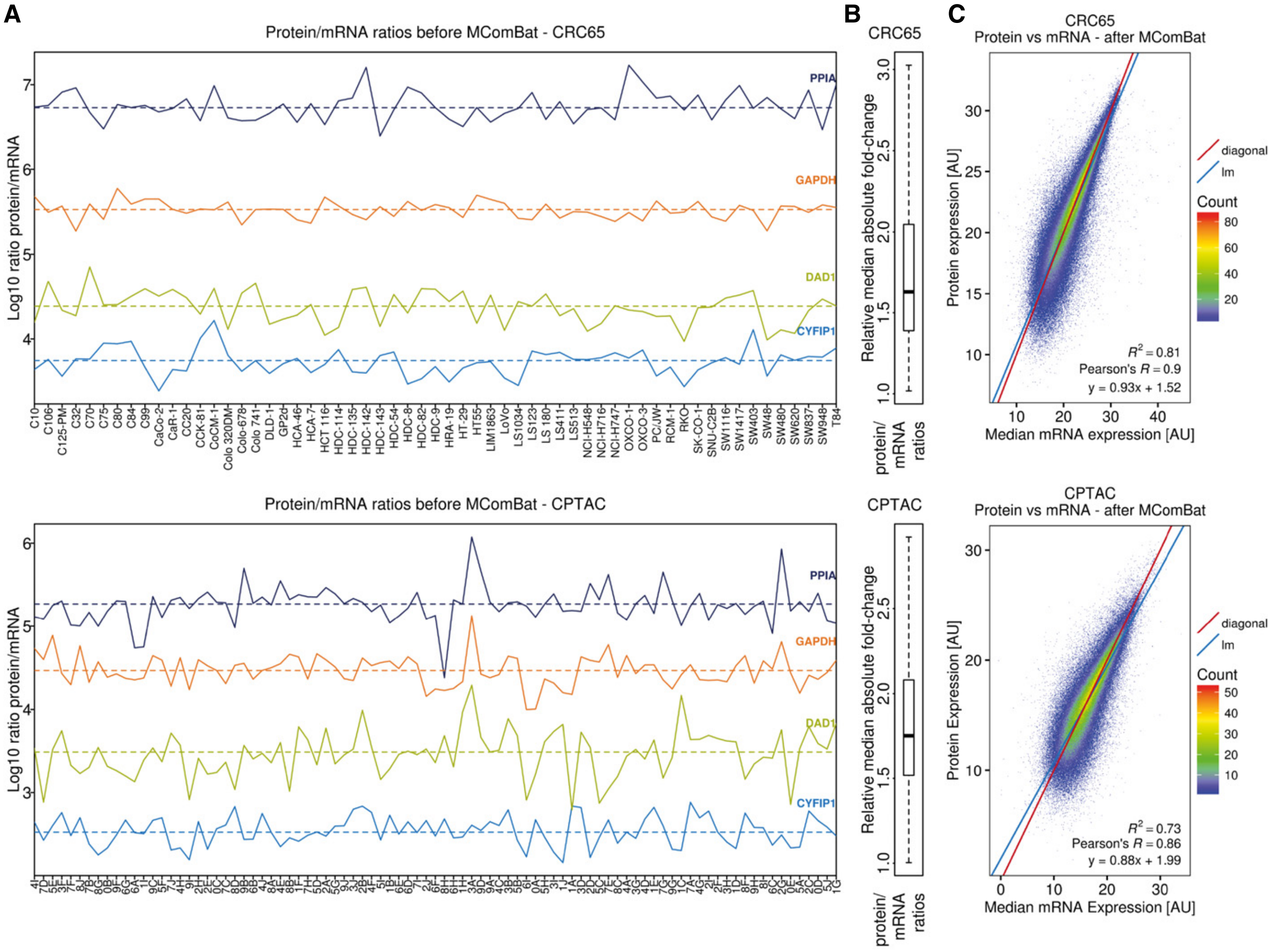

Figure EV3. Estimating protein levels from mRNA levels (related to Fig 3).

A Protein/mRNA ratios (log10) for four proteins plotted across the CRC65 and CPTAC datasets show that this ratio is relatively stable for a given protein/mRNA.

B Distribution of the median absolute fold-change in protein/mRNA ratios, relative to the protein-wise median protein/mRNA ratio, indicating that variation in the protein/mRNA ratios was typically below twofold. The median is marked with a bold horizontal line inside a box spanning the interquartile range (IQR) from the 25% quantile ($q_{25\%}$; lower horizontal line) to the 75% quantile ($q_{75\%}$; upper horizontal line), while the whiskers extend to $a = q_{25\%} - 1.5 \times \text{IQR}$ and $b = q_{75\%} + 1.5 \times \text{IQR}$. Outliers according to the standard boxplot definition ($x < a$, as well as $x > b$) were excluded for visual clarity.

C Scatterplot of median mRNA expression after MComBat adjustment for all transcripts across all cell lines/tumours versus protein expression in the CRC65 and CPTAC datasets, indicating that protein levels can be estimated reasonably well from transcript levels (lm: linear model; R^2: coefficient of determination; see main text and Appendix Supplementary Methods for details).

 © 2017 The Authors

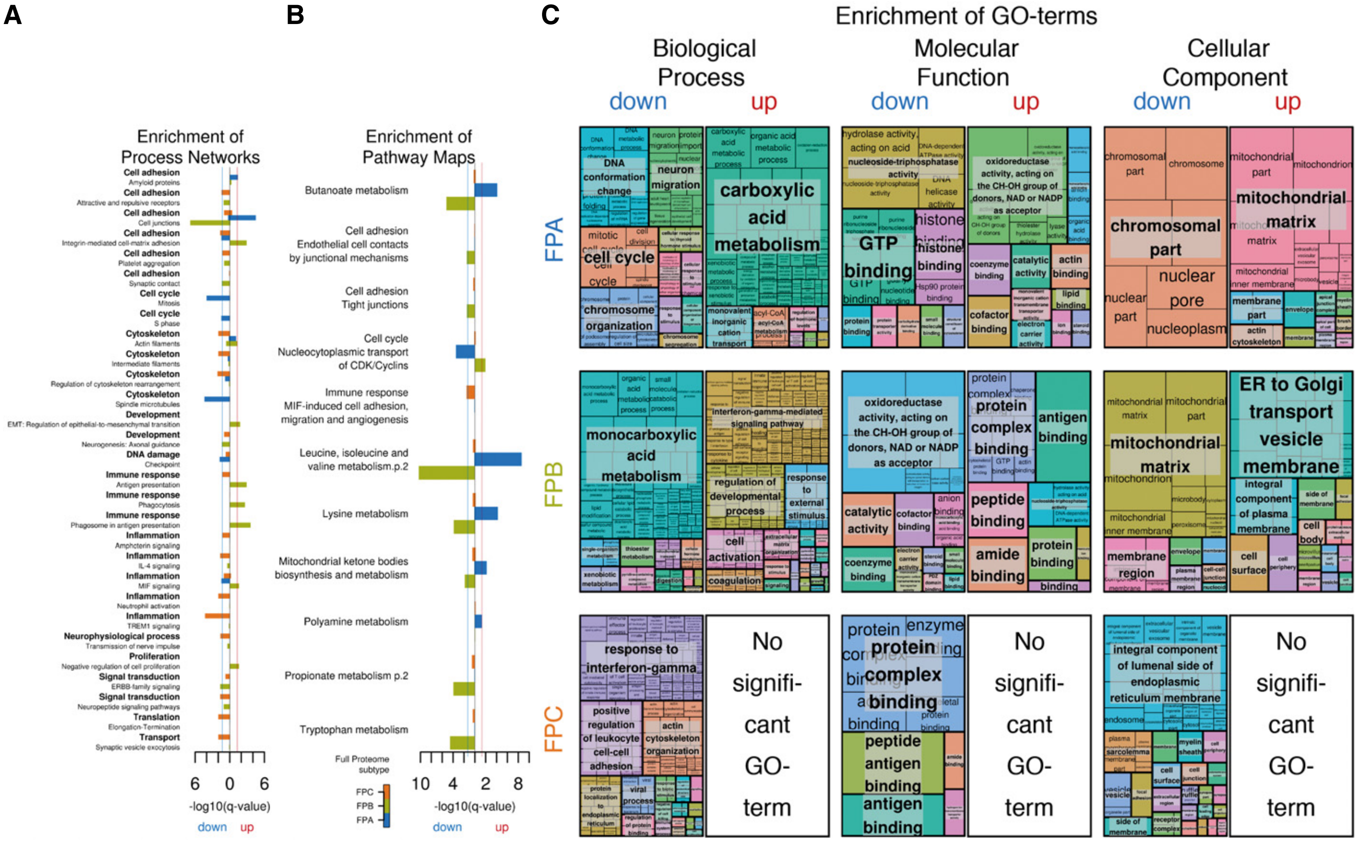

Figure EV4. Enriched Process Networks, Pathway Maps & GO terms (related to Fig 3).

A Bar chart visualising the negative logarithm to the base of ten with respect to the enrichment q-value of process networks significantly (minimum q-value ≤ 0.05, MetaCore) enriched in proteins down-regulated (to the left of the origin) or up-regulated (to the right of the origin) in the different FPSs.

B Same as (A) for pathway maps.

C Treemaps generated from R scripts as downloaded from REViGO after summarisation of GO terms significantly (minimum q-value ≤ 0.05, MetaCore) enriched in proteins down- or up-regulated in the different FPSs. See Appendix Supplementary Methods for details.

© 2017 The Authors

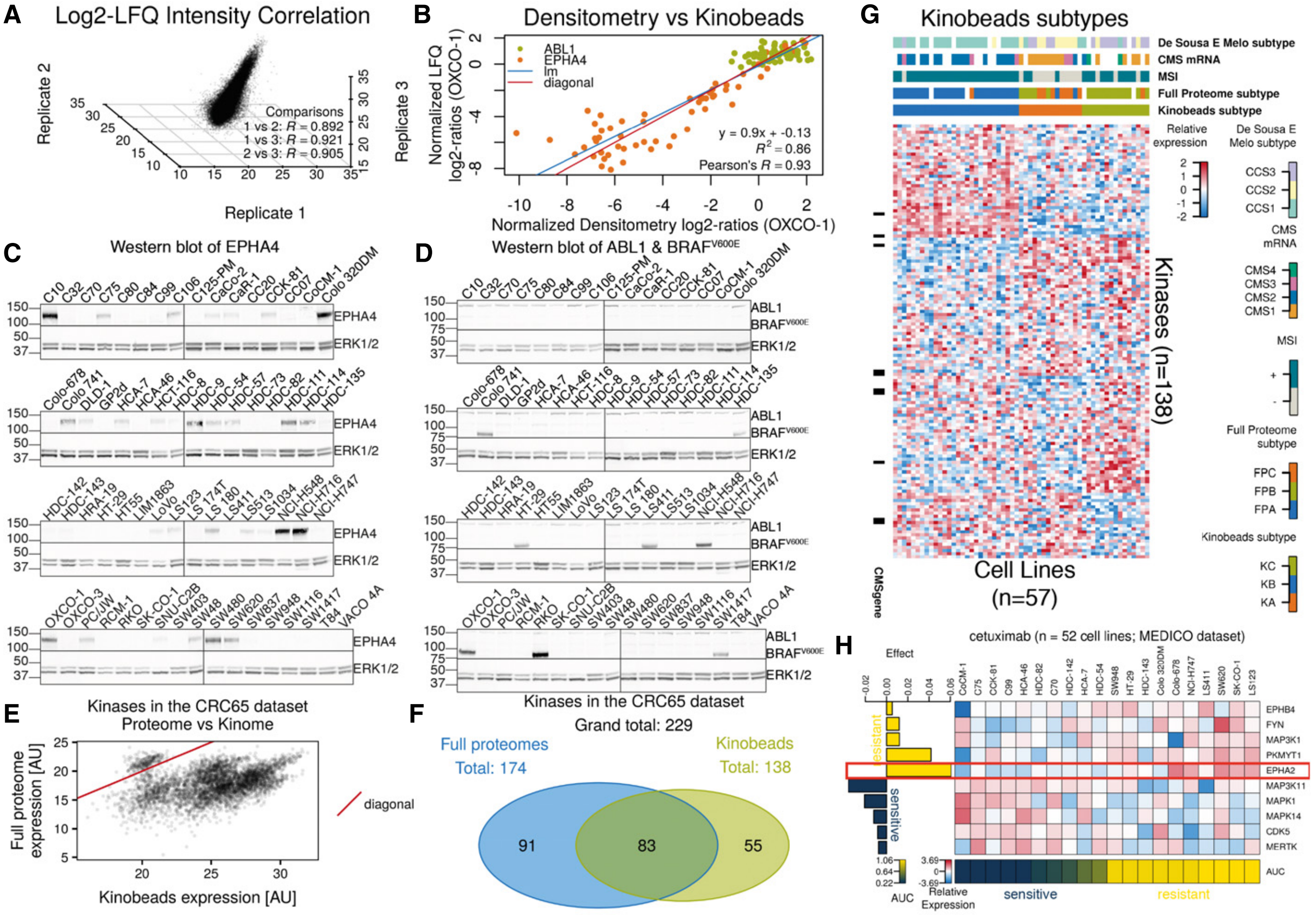

Figure EV5. Reproducibility of Kinobeads-based quantification, Western blots of EPHA4, ABL1 and BRAF^{V600E}, correlation of LFQ intensity with densitometry and full proteome giBAQ values, as well as overlap of kinase IDs, Kinobeads Subtypes and predictive markers for cetuximab (related to Fig 4).

A 3-D scatterplot showing the correlation of LFQ values for all proteins across all cell lines in the three biological replicates of the Kinobeads pulldowns after ComBat adjustment (R: Pearson's R).

B–D Scatterplot (B) and underlying Western blot data (C and D) visualising the correlation between normalised densitometry log2-ratios relative to C10 and normalised LFQ log2-ratios relative to OXCO-1 for EPHA4 and ABL1 (diagonal: $x = y$; lm: linear model; R^2: coefficient of determination). Densitometry was performed using ImageStudioLite v5.2.5 (LI-COR), expressing EPHA4 and ABL1 expression relative to the respective ERK1/2 signal, followed by dividing all expression values by the expression value of OXCO-1 (present on each gel) and log2-transformation. LFQ log2-ratios were based on log2-transformed and median-centred LFQ intensities after ComBat adjustment. We reverted the log2-transformation and divided all LFQ values by the LFQ value of OXCO-1, followed by log2-transformation of the resulting ratios. Cell lines harbouring the BRAF^{V600E} mutation are visualised using a mutation-specific antibody.

E Scatterplot of kinase expression as quantified using CRC65 full proteome measurements versus Kinobeads experiments. Kinase expression is systematically higher in the Kinobeads data.

F Venn diagram of kinase identifications in the CRC65 full proteome and Kinobeads experiments. The two datasets are relatively complementary to each other. The datasets were colour-coded as in the main manuscript (green = Kinobeads, blue = CRC65 full proteomes).

G Heat map of standardised, log2-transformed and median-centred LFQ kinase quantification (z-scores) across the CRC65 dataset. Cell lines are displayed as columns and kinases are shown as rows (kinases measured in at least two biological replicates across at least one-third of the cell lines were used to find subtypes). Black bars to the left of the heat map indicate the presence of the respective kinase in the CMS classifier. Annotation bars on top of the heat map visualise the membership of the different cell lines/patients in five annotation categories. Columns are ordered hierarchically according to the consensus clustering results, while rows are ordered according to their association with a particular KS (see Appendix Supplementary Methods).

H Effect-size heat map of cetuximab visualising EPHA2 as a robust predictive marker of resistance.

 © 2017 The Authors

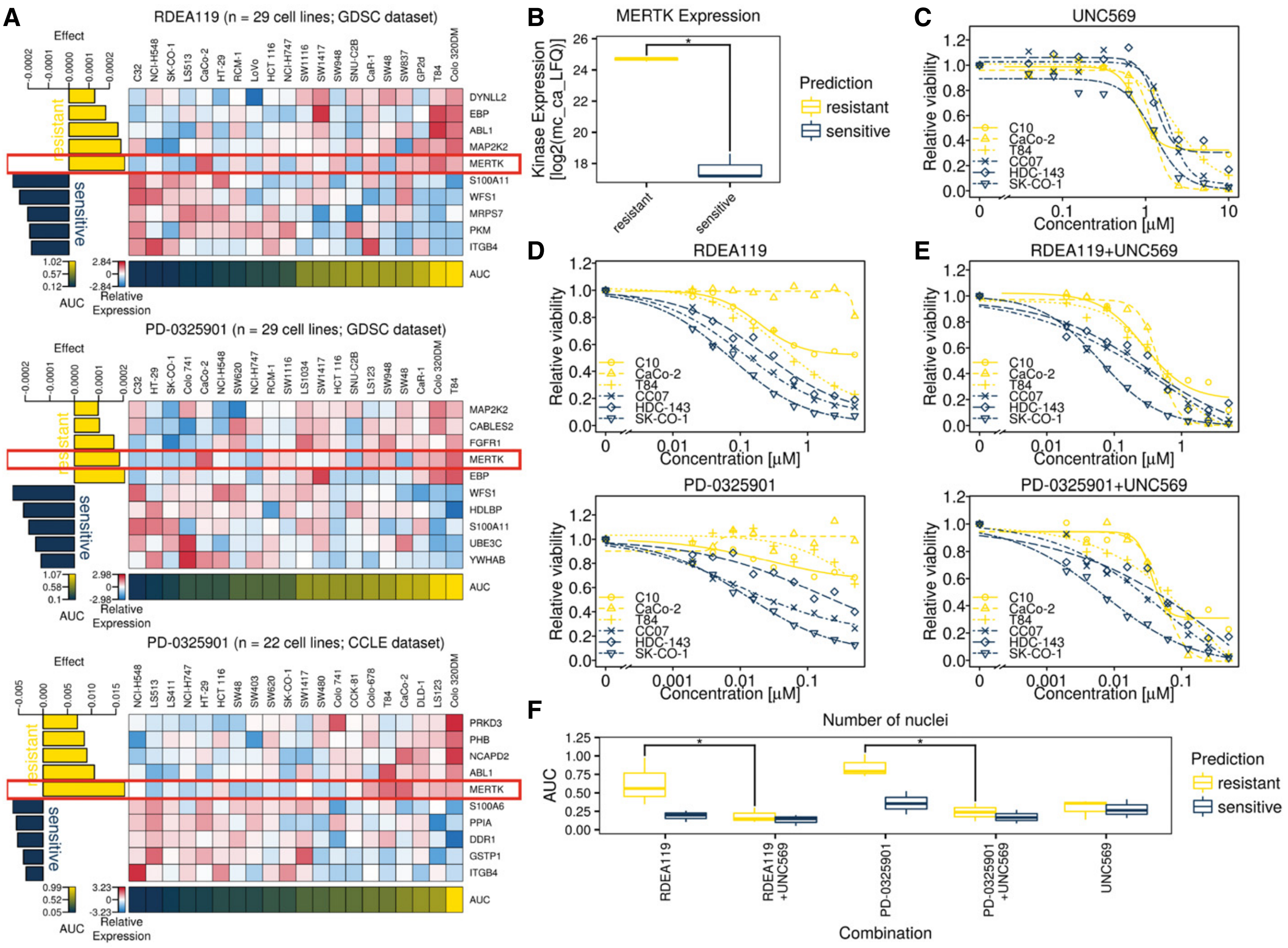

Figure EV6. Co-treatment with UNC569 (MERTK inhibitor) and MEK inhibitors is more effective than treatment with MEK inhibitors alone (related to Fig 5).

A Effect-size heat maps of two drugs (one from two datasets) targeting MEK1/2 show consistent association of high MERTK expression with drug resistance, even when drug sensitivity is modelled based on all proteins in the Kinobeads expression matrix (see Appendix Supplementary Methods).

B Boxplots of MERTK expression (log2-transformed and median-centred LFQ values after ComBat adjustment) in cell lines predicted to be sensitive (CC07, HDC-143, SK-CO-1; dark blue) or resistant (C10, CaCo-2, T84; yellow) towards two MEK1/2 inhibitors RDEA119 and PD-0325901. The whiskers extend to the minimum and maximum expression of MERTK for cell lines with a given sensitivity prediction, while the median expression of MERTK is marked with a bold horizontal line inside a box spanning the interquartile range (IQR) from the 25% quantile (lower horizontal line) to the 75% quantile (upper horizontal line). Resistant cell lines show significantly higher expression of MERTK than sensitive ones ($P \leq 0.05$, one-sided Mann–Whitney test).

C–E 10-point dose–response curves of (C) the MERTK inhibitor UNC569, (D) two MEK1/2 inhibitors RDEA119 and PD-0325901 and (E) their constant-ratio combination (see Appendix Supplementary Methods) for the same cell lines as in (B) from our own *in vitro* experiments. Relative response is expressed as the mean of three technical replicates.

F Boxplots of the AUC from (C–E), showing significant differences between cell lines predicted to be sensitive (CC07, HDC-143, SK-CO-1; dark blue) and cell lines predicted to be resistant (C10, CaCo-2, T84; yellow) to RDEA119 and PD-0325901 ($P \leq 0.05$, one-sided Mann–Whitney test). The whiskers extend to the minimum and maximum AUC for a given drug and sensitivity prediction, while the median AUC is marked with a bold horizontal line inside a box spanning the interquartile range (IQR) from the 25% quantile (lower horizontal line) to the 75% quantile (upper horizontal line). This difference disappears when co-treating cells with UNC569 in addition to MEK1/2 inhibitors.
